# Surgical Treatment of Periarticular Distal Radius Fracture in Elderly: A Systematic Review

**DOI:** 10.3390/medicina60101671

**Published:** 2024-10-11

**Authors:** Gianluca Testa, Flora Maria Chiara Panvini, Marco Simone Vaccalluzzo, Andrea Giovanni Cristaudo, Marco Sapienza, Vito Pavone

**Affiliations:** Department of General Surgery and Medical Surgical Specialties, Section of Orthopedics and Traumatology, A.O.U. Policlinico Rodolico-San Marco, University of Catania, Via Santa Sofia 78, 95123 Catania, Italy; gianpavel@hotmail.com (G.T.); marcovaccalluzzo@hotmail.it (M.S.V.); cristaudoandrea@gmail.com (A.G.C.); marcosapienza09@yahoo.it (M.S.); vitopavone@hotmail.com (V.P.)

**Keywords:** distal radius fracture, elderly, aging fracture, ORIF, plate, K-wires, external fixation, osteoporosis, DRF, surgical treatment

## Abstract

*Background/Objectives*: The treatment of periarticular distal radius fractures remains challenging. Different surgical treatment options have been proposed as alternatives to conservative treatment. This systematic review aims to compare the functional outcomes, radiological outcomes, and complications among volar locking plates (VLPs), Kirschner-wire fixations, and external fixations (EFs) for distal radius fractures in patients aged 60 years and older. *Methods*: We conducted a comprehensive search of PubMed, Cochrane, and Science Direct databases assessing the effects of VLP, EF, and K-wire treatments for distal radius fractures in patients aged 60 years and over. The primary outcome was the evaluation of the range of motion (ROM) degrees after three surgical procedures, trying to assess the best treatment option. The secondary outcome included evaluation of the Disabilities of the Arm, Shoulder, and Hand (DASH) score, quick-DASH score, Patient-Rated Wrist Evaluation (PRWE) score, Visual Analog Scale (VAS) score, grip strength, radiographic assessment, and complications comparing VLPs, EFs and K-wires. *Results*: A total of 23 studies were included, comparing VLP, EF, and K-wire fixation. The overall population comprised 5618 patients, with 4690 females and 1015 males, of which 4468 patients were treated with VLP, 503 with EF, and 647 with K-wire. The most common complications among the VLP group were complex regional pain syndrome (7.5%) and carpal tunnel syndrome (6.8%); for the EF group, infections (9.8%) and carpal tunnel syndrome (6.8%); and for the K-wire group, carpal tunnel syndrome (7.5%) and infections (6.9%). *Conclusions*: VLP showed better clinical outcomes in the first few months after treatment. However, these differences decreased over time and became similar after one year. EF and K-wire fixations remain easier to manage during surgery.

## 1. Introduction

Distal radius fractures (DRFs) represent one of the most common skeletal injuries, particularly among the elderly population, in which they frequently result from low-energy trauma, such as falls. These fractures account for up to 18% of all fractures in individuals aged 65 years and older [1]. As the global population ages, the incidence of these fractures is anticipated to increase, posing significant challenges in their management and rehabilitation [1]. DRFs not only impair wrist function but also have substantial impacts on the quality of life of the elderly, potentially leading to long-term disability and increased dependency [2].

The management of periarticular DRFs in elderly patients is particularly challenging due to the presence of comorbidities and the decreased bone quality often associated with osteoporosis, which complicate the healing process [1]. While conservative treatments, such as casting, have traditionally been employed, surgical intervention has become increasingly favoured because of its potential to provide better stabilisation and facilitate earlier functional recovery [2]. However, despite the variety of surgical techniques available, including volar locking plates (VLPs), Kirschner-wire fixation (K-wire), and external fixation (EF), there remains a lack of consensus on the optimal approach for this patient population [3].

Each of these surgical techniques has its own advantages and challenges. Volar locking plates (VLPs) are widely used because of their ability to provide stable fixation and to allow for early mobilisation, although they are not without risks, such as soft tissue complications [2,3]. Kirschner-wire fixation (K-wire), while minimally invasive and quicker to perform, may offer less stability, particularly in more complex fracture patterns. External fixation (EF) is advantageous for maintaining reductions in comminuted fractures but could carry a higher risk of complications [2,3].

Given the diversity of surgical options and the ongoing debate regarding their relative effectiveness, there is a critical need for a systematic evaluation of these approaches. This systematic review aims to compare the functional outcomes, radiographic results, and complication rates associated with VLPs, K-wires, and EFs in the treatment of periarticular DRFs in elderly patients. By synthesising the available evidence, this study seeks to provide clearer guidance for clinical practice and contribute to the development of more standardised treatment protocols for this increasingly prevalent condition.

## 2. Materials and Methods

### 2.1. Study Selection

In accordance with the guidelines of the Preferred Reporting Items for Systematic Reviews and Meta-Analysis (PRISMA) [4], the PubMed, Cochrane, and Science Direct databases were systematically reviewed by two independent authors (F.M.C.P. and A.G.C.). Five independent strings were used, as follows: “((distal radius fracture) AND (wire) OR (pin) AND (elderly))” and “((distal radius fracture) AND (external fixation) AND (elderly))” and “((distal radius fracture) AND (surgical treatment) AND (elderly))” and “((distal radius fracture) AND (plate) AND (elderly))”. From each included original article, a standard data entry form was utilised to extract the number of patients, type of study, treatment, follow-up, and year of the study.

The quality assessments of the studies were performed in duplicate by two independent reviewers (F.M.C.P. and A.G.C.). Conflicts regarding data were resolved via consultation with a senior surgeon (G.T.).

### 2.2. Inclusion and Exclusion Criteria

Eligible studies for this systematic review included surgical treatment for DRFs, including K-wire, EF, and plate and screw fixations of periarticular fractures of the wrist, in patients aged over 60 years old, with a follow-up period of at least six months and clinical outcomes reported at the medium and final follow-ups. Articles in the English language were selected, evaluating those published from January 2013 to March 2024. All articles found in the literature that focused on the main topic but published in other languages or before 2013 were excluded. Patients treated with casts or studies in which clinical follow-up was not evaluated, as well as those that involved pathological fractures, were excluded. Articles including both younger and older populations were selected only if separate outcomes between the two groups were made explicit, allowing for the outcomes in only those over 60 years of age to be considered.

### 2.3. Risk of Bias Assessment

The data extracted from the studies included author, country, year of publication, study type, sample size, demographics, surgical procedure, and clinical follow-up. Bias was assessed using the Risk of Bias in Non-Randomised Studies of Interventions (ROBINS-I) tool [5]. Two authors (F.M.C.P. and A.G.C.) performed the evaluations independently. Any discrepancies were discussed with the senior investigator (G.T.) for the final decision.

## 3. Results

### 3.1. Included Studies

**Figure 1:**
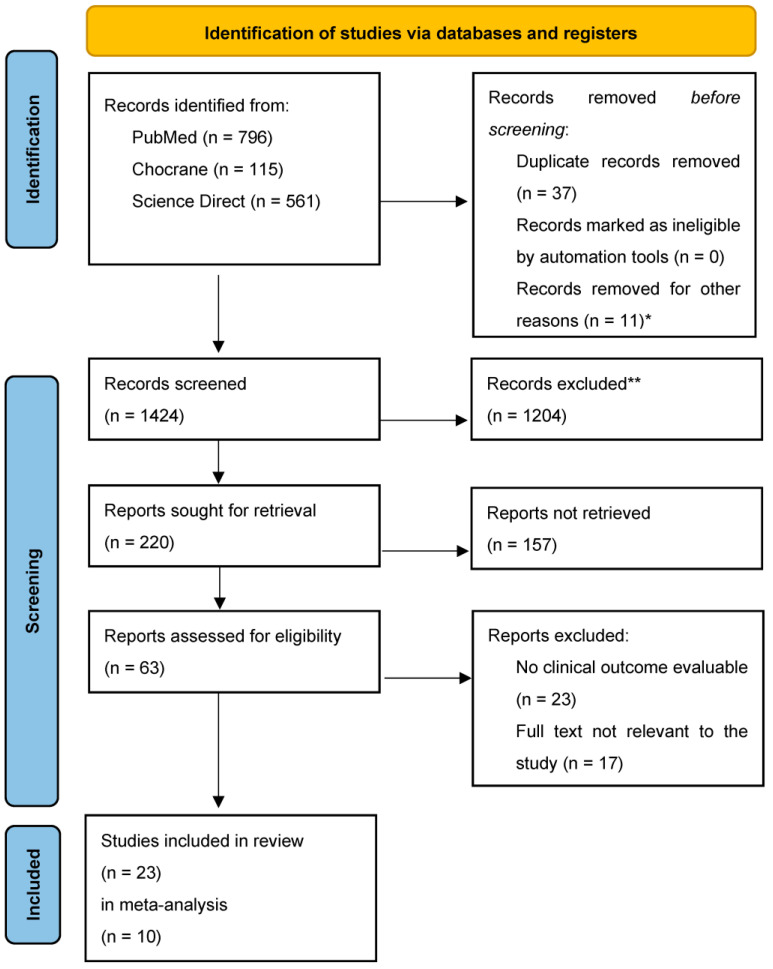
PRISMA 2020 flow diagram for new systematic reviews. * Unsuitable due to unassessable abstracts or inability to access the full text. ** Unsuitable according to the search criteria.

A total of 1472 studies were identified. Our study focused on recent research (from January 2013 to March 2024) on the treatment of DRFs. After the exclusion of 37 duplicate articles, 11 were determined as unsuitable due to unassessable abstracts or an inability to access the full text. Upon further analysis, 1204 articles were excluded for not aligning with the main topic or failing to meet the predefined inclusion criteria. An additional 157 articles were excluded because of incompatible abstracts, leaving 63 articles for full-text reading. Finally, 23 articles were eligible for the systematic review. All articles selected were RCTs and retrospective studies [6,7,8,9,10,11,12,13,14,15,16,17,18,19,20,21,22,23,24,25,26,27,28]. This selection process is illustrated by the PRISMA flow diagram shown in Figure 1.

The main findings of the included articles are summarised in Table 1.

Out of the 23 selected studies, 22 addressed distal radius fractures treated with VLP, 8 with EF, and 7 with K-wire (Table 1).

The overall population included a total of 5618 patients, with 4690 females and 1015 males; unfortunately, two studies did not report gender data [6,7]. Of these patients, 4468 were treated with VLP, 503 with EF, and 647 with K-wire.

The most frequently reported outcomes such as ROM, radiological outcomes, DASH score, quickDASH score, VAS score, PWRE score, and grip strength were then analysed.

### 3.2. Range of Motion Analysis

Although there was some variability in the study designs, patient demographics, and follow-up durations across the studies, the overall trends in ROM recovery remained consistent. This consistency reinforces the reliability of the results, supporting the use of these surgical techniques in clinical practice despite the moderate heterogeneity observed.

Zhang et al. [8] compared VLPs and EFs. Better flexion results were found in the EF group (65.3° ± 6.1° vs. 63.9° ± 8.2°), while the VLP group had better results in extension and pronation (61.3° ± 5.5° vs. 55.3° ± 9.2° and 81.2° ± 6.3° vs. 78.6° ± 4.7°, respectively). Yu Yi Huang et al. [9] found better results for VLP synthesis in full ROM recovery instead of the EF group (flex: 65.2° ± 7.6°, ex: 61.1° ± 11.6°, supination: 80° ± 7.2°, and pronation: 82.5° ± 8.0° vs. 61.9° ± 10.0°, 58.6° ± 7.7°, 74.7° ± 6.6°, and 78.5° ± 8.6°, respectively). Forearm supination was significantly better in patients treated with a VLP (*p* = 0.002). In accordance, different authors showed similar outcomes for VLPs [10,11,12,13,14,15].

Additionally, Bartl et al. [16] reported the differences between pre- and post-treatment with a plate and screws (extension: 7.5° ± 11.7°, flexion: 8.2° ± 11.9°, supination: 2.5° ± 5.9°, and pronation: 2.8° ± 5.6°). Yalin et al. [17] did not find any statistical difference among the ROM recoveries in the three groups (VLP, EF, and K-wire). On the other hand, Chung et al. [18] found slightly better outcomes for extension in the VLP compared to the EF and K-wire groups. The same results were reported by Goehre et al. [19], who compared VLPs and K-wires, with plate and screws fixation seeming to lead to better outcomes. Despite other authors, Yigit et al. [7] noted better results for flexion and extension in the K-wire group (64.6° ± 8° and 61.5° ± 6.5° vs. 64.5° ± 7.2° and 60.5° ± 5.3°), while the VLP group had better results for pronation and supination at 6- and 12-month follow-ups.

### 3.3. Radiological Outcome

Zhang et al. [8] compared results between a VLP group and an EF group and found that at the last follow-up, the radiographic parameters were better in the VLP group than in the EF group (*p* < 0.05). Similarly, the results of Huang et al. [9] were significantly more acceptable in the VLP group (*p* <0.05). On the other hand, Yigit et al. [7] compared the results between the VLP group and the K-wire group using postoperative images taken at 1-year follow-up and found no significant differences between the two groups in radial inclination (*p* = 0.975), radial tilt (*p* = 0.661), and radial height (*p* = 0.346). Avci et al. [20] compared a VLP group and a K-wire group, revealing significant differences between them in favour of the first one regarding radial height, volar tilt, radial inclination, and joint stepping (*p* < 0.001). Chung et al. [18] compared the radiological outcomes in three groups, analysing the volar/dorsal tilt, ulnar variance, radial inclination, and radial height, with the VLP group showing better results (3° (range: 0–6); 1.4 mm (range: 0.7–2.0); 22° (range: 20–23); and 11.1 mm (range: 10.1–12.1), respectively).

Most authors analysed only the treatment of distal wrist fractures with plates and screws. Similar results were found for radial inclination, radial height [6,10,11,13,21,22,23], radial tilt [10,11,13,21,22], and ulnar variance and articular step-off [6,10,13,21,23].

### 3.4. Clinical Outcome

**Figure 2:**
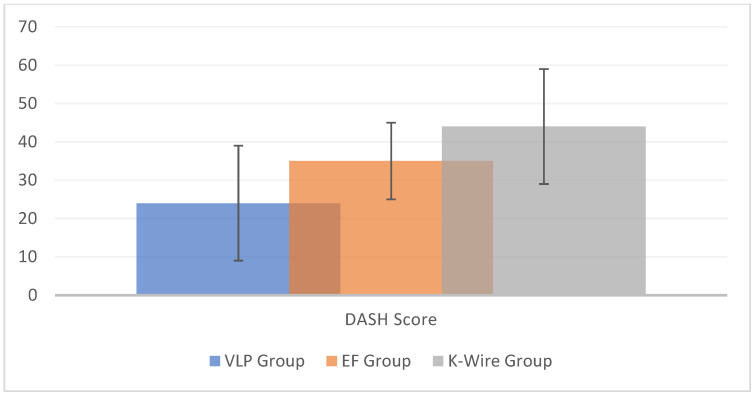
Histogram of the DASH scores, considering all available data. The vertical lines represent the standard deviations. Eight studies reported DASH score data for a VLP group, two studies for an EF group, and one study for a K-wire group.

One of the clinical outcomes analysed was the DASH score (Figure 2). Zhang et al. [8] compared the DASH score between a VLP group and an EF group and found no significant difference in the overall DASH scores (*p* = 0.25). Yalin et al. [17] compared the DASH scores in a VLP group, EF group, and K-wire group, with the EF group showing better results (47 ± 4.49, K-wire: 43.85 ± 3.87, and VLP group: 44.32 ± 4.24). Similar DASH score results were reported for the VLP group [6,10,11,15,16,24].

**Figure 3:**
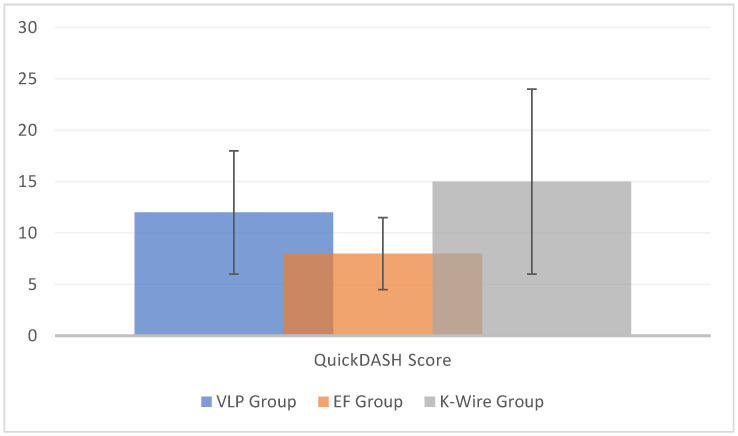
Histogram of the quickDASH scores, considering all available data. The vertical lines represent the standard deviation. Seven studies reported quickDASH score data for a VLP group, two studies for an EF group, and one study for a K-wire group.

QuickDASH scores were also evaluated (Figure 3). Kaya et al. [25] compared a VLP group and an EF group, with the former showing better results (25.71 ± 22 vs. 24.42 ± 22.71). There was no statistical difference between the groups in terms of clinical scores (*p* > 0.05). Otherwise, Avci et al. [20] compared a VLP group and a K-wire group, with the K-wire group showing better results (12.75 ± 6.84 vs. 9.77 ± 6.63).

The plate and screw technique results were not completely homogeneous [13,21,23,26]. On contrary, similar outcomes were found when evaluating QuickDASH scores for bridged and non-bridged external fixators [27].

**Figure 4:**
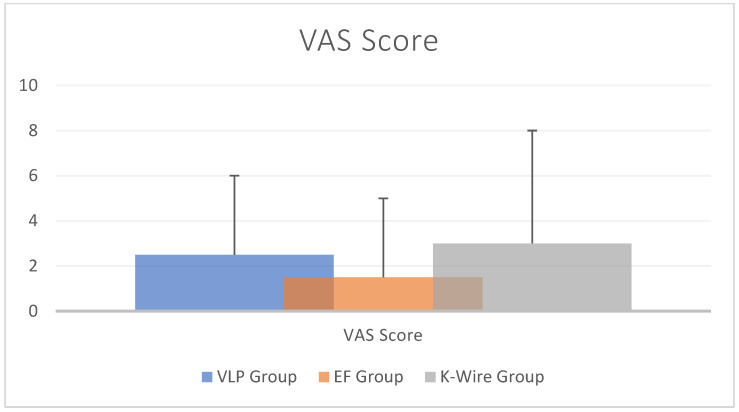
Histogram of the VAS score, considering all available data. The vertical lines represent the standard deviation. Five studies reported VAS score data for a VLP group, two studies for an EF group, and two studies for a K-wire group.

VAS scores were also evaluated (Figure 4). Yigit et al. [7] compared a VLP group and K-wire group, showing no significant differences between 6 months and 1 year (*p* = 0.14 and *p* = 0.95). Similarly, Avci et al. [20] compared a VLP group and a K-wire group and found no difference regarding the VAS scores at the last follow-up. Also, Kaya et al. [25] did not find a statistical difference between groups (*p* > 0.05).

PWRE scores were also analysed. Kaya et al. [25] compared a VLP group and an EF group, with the mean PRWE being 27.14 ± 25.2 in the VLP group and 31.46 ± 21.34 in the EF group. However, Yigit et al. [7] compared a VLP group and K-wire group and found no significant differences in the PRWE scores between 6 months and 1 year at any time point (*p* > 0.05).

Open reduction and internal fixation (ORIF) had similar positive results for PRWE and VAS scores in the literature [10,11,13,15,22,23,24,26].

Grip strength was shown to have no significant differences between VLP and K-wire groups [19], except for Chung et al. [18], who found that the VLP group had better results (84%).

### 3.5. Complications

**Figure 5:**
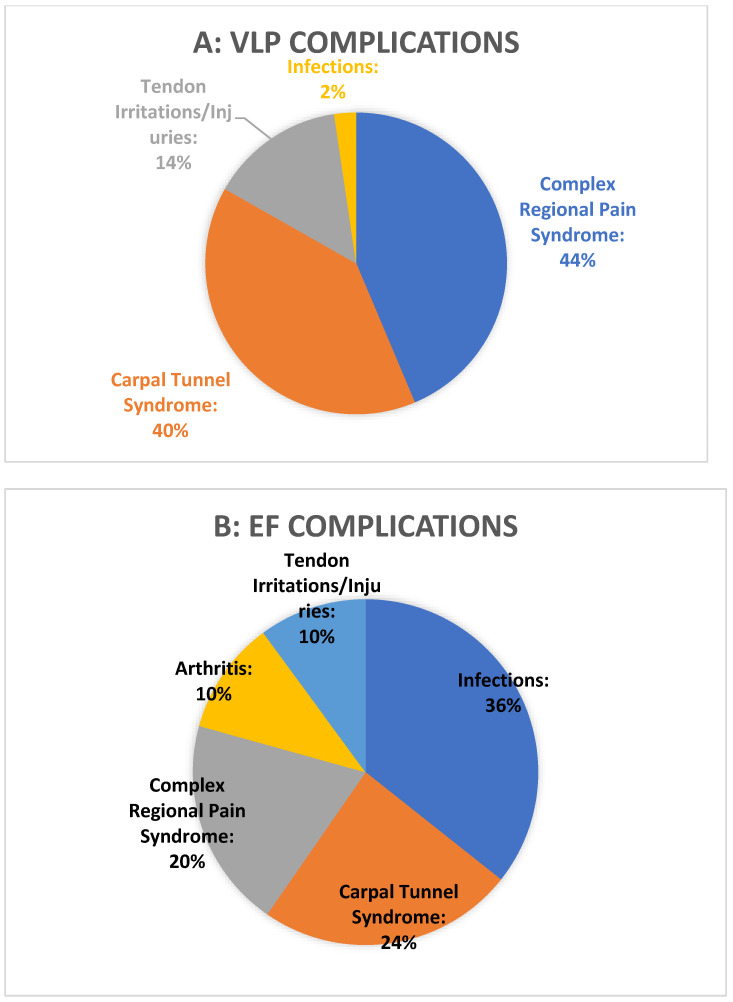

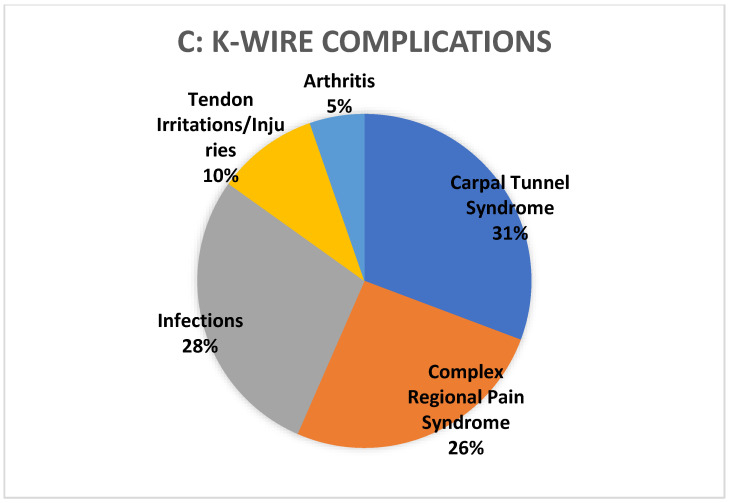
(**A**) Pie diagram representing complications arising from surgery with VLP. (**B**) Pie diagram representing complications arising from surgery with EF. (**C**) Pie diagram representing complications arising from surgery with K-wire.

With two studies not reporting data on complications, the VLP group had a total of 4393 patients, the EF group had 478, and the K-wire group had 634. The most frequently encountered complications were arthritis, infections, tendon irritation/injuries, carpal tunnel syndrome, and complex regional pain syndrome. Less frequent complications included loss of reduction, implant malposition, malunion, stiffness, scar adherence, and others. Complex regional pain syndrome was the most reported complication, with 331/4393 (7.5%) in the VLP group, 26/478 (5.4%) in the EF group, and 40/634 (6.3%) in the K-wire group. Carpal tunnel syndrome was observed in 299 cases (6.8%) for the VLP group, 32 cases (6.6%) for the EF group, and 48 cases (7.5%) for the K-wire group. Postoperative arthritis was observed in 17 patients (0.3%) for the VLP group, 14 (2.9%) for the EF group, and 8 (1.3%) for the K-wire group. A total of 18 cases (0.4%) of infection presented in the VLP group, 47 cases (9.8%) in the EF group, and 44 cases (6.9%) in the K-wire group. Tendon irritation/injuries were considered as a single group and were found in 109 cases (2.48%) in the VLP group, 13 cases (2.78%) in the EF group, and 15 cases (2.4%) in the K-wire group. The three different complication rates are presented in Figure 5.

## 4. Discussion

The treatment of distal radius fractures remains controversial. Different options have been analysed in the literature, and sometimes the choice depends on several factors. It is essential to consider patient characteristics, activity requirements, fracture stability and displacement to make an informed decision and choose the most appropriate approach [25]. In addition, in clinical practice, many patients are unwilling to tolerate the discomfort associated with casting and long-term immobilisation, leading them to forgo conservative treatment with a cast. This is particularly true for patients with type C distal radius fractures who are seeking rapid pain relief and a reduction in the risk of complications, such as malunion and post-traumatic arthritis, among others. As a result, both clinicians and patients often opt for surgical treatment to accelerate the healing process [8], restore articular congruity, improve clinical outcomes, and achieve normal wrist function. In the elderly population, this relationship between the resolution of articular incongruity and improved clinical outcomes is not yet clear. In contrast, in younger individuals, it is well documented that inadequate reduction is strongly correlated with unsatisfactory clinical outcomes. There is no single surgical approach or type of fixation recommended for all fractures; therefore, surgeons must be familiar with all methods and select the appropriate fixation method based on the fracture type [25].

No operative method has been accepted yet as the standard of care. It is generally accepted that stable and non-displaced fractures can be treated conservatively with good anatomical and functional outcomes. Several authors have shown that conservative treatment is often associated with secondary loss of reduction, but good outcomes can still be achieved [19].

It is generally accepted that simple extra-articular distal radius fractures are treated with closed reduction and percutaneous Kirschner wire fixation or external fixation, whereas multifragment intra-articular distal radius fractures are treated with open reduction and volar or dorsal locking plates [7]. Patients over 65 years of age treated with both VLP and K-wire have comparable functional outcomes at one year [19]. An advantage is observed in the first few months after plate fixation, with patients able to resume daily activities four weeks earlier than those treated with Kirschner wire fixation. This may be due to the primary stability of the osteosynthesis. VLP offers several advantages, such as direct fracture visualisation, stable fixation, subchondral support, subsequent articular surface correction, and early motion. However, with an average operative time of 23 min, Kirschner wire fixation requires only about one-third of the operative time required for plate fixation (median: 60 min). Otherwise, Kirschner wire fixation is a minimally invasive alternative with comparable clinical outcomes [19].

External fixation appears to have a significantly shorter operative time and less intraoperative blood loss compared to VLP (*p* < 0.001) and avoids the problem of a second surgery to remove the implant. The advantages of EF over VLP, such as less bleeding, shorter operative time, and no incision, are in line with the principles of modern minimally invasive surgery [8]. No significant differences were found between VLP and K-wire treatments for the VAS scores, PWRE scores, and wrist ROM at 6 months and 1 year. Initially, the VLP group had better ROM and VAS scores compared to the K-wire group; postoperative recovery was faster in the plate group. The difference in the PWRE scores in the K-wire group tended to decrease over time. The lower PWRE and VAS scores in the K-wire group may be due to a delayed initiation of wrist ROM exercises. The K-wire technique has advantages such as minimal blood loss, shorter operative time, and minimal wound infection in elderly patients [7].

Despite the good clinical results of the other techniques, the VLP group seems to have relatively better imaging data and wrist joint activity at long-term follow-up [8].

The restored wrist motions seem to be quite similar, with greater wrist extension and pronation of the forearm in the VLP group compared to the EF group and slightly better supination of the forearm. The wrist flexion was slightly better in the EF group [8].

All techniques have some complications and although K-wire fixation and EF have a generally higher risk of infection and postoperative arthritis, complex regional pain syndrome and carpal tunnel syndrome are more common with VLP treatment [7,25]. Otherwise, complications are rarely observed.

As an alternative to the previously described methods of fixation in cases of non-comminuted or one- and two-fragment volar or dorsal-displaced wrist fractures, one might consider Epibloc elastic–dynamic fixation, associated with an intrafocal pinning reduction in elderly patients [29].

Our results should be interpreted considering some limitations. First, there is heterogeneity in the types of studies included, as both randomised controlled trials (RCTs) and retrospective studies were included. In addition, the literature contains significantly more studies of treatment with plates and screws than with external fixation and K-wire. Therefore, more plate and screw studies were selected, resulting in a larger sample sise for the VLP group compared to the EF and K-wire groups.

## 5. Conclusions

In conclusion, VLP demonstrated superior outcomes for range of motion and clinical outcomes in the initial postoperative period, making it the optimal treatment when early postoperative functional recovery is paramount. It facilitates a faster return to daily activities. However, its differences with other surgical techniques (EF and K-wires) tended to decrease over time, becoming comparable at one year of follow-up, possibly due to good adherence to physiotherapy, which can increase with the improvement in clinical outcomes. While the advantages of EF and K-wires, such as reduced bleeding, shorter surgical times, and absence of incisions, allow for better operative management, the indications for the various treatment modalities should be evaluated based on the risk–benefit ratio in patients over 65 years of age with distal radius fractures.

## Figures and Tables

**Table 1:** Summary of the twenty-three articles eligible for the systematic review (RS: retrospective study; HCS: historical cohort study; months: m; years: y).

Authors	N°Pz	Surgical Techniques	M/F	Age	Study Design	Follow-Up	Treatment Results
Ozcan Kaya et al. [25] (2022)	27	VLP (14), EF (13)	16F, 11M	VLP 64.21 ± 3.06 EF 67.69 ± 5.67	RS	VLP: 31.64 m ± 35.7 EF: 28.84 m ± 12.7	Mean Q-DASH score: 25.7 (VLP) and 24.4 (EF). Mean PRWE: 27.1 (VLP) and 31.4 (EF). No statistical differences in clinical scores and complications.
He Zhang et al. [8] (2023)	62	EF (30), VLP (32)	49F, 13M	EF 73 ± 6 VLP 72 ± 7	RS	6 m	EF group: decreases in operative time, intraoperative blood loss, injury-to-surgery time, and hospital stay. VLP: Better radiographic parameters and wrist joint function. No significant difference in overall DASH score and complications.
Seyhmus Yigit et al. [7] (2020)	72	VLP (38), K-wire (34)	72F	VLP 70.4 ± 6.6 K-wire 70.7 ± 7.17	RS	12 m	No statistical differences between VLPs and K-wires at 1-year follow-up.
Ozgur Avci et al. [20] (2023)	68	VLP (31), K-wire (37)	46F, 22M	VLP 79.00 ± 2.92 Wire 77.89 ± 2.25	RS	VLP 60.19 ± 30.63 Wire 65.46 ± 25.96	Similar clinical outcomes at last follow-up. Statistically significant differences in terms of radiological outcomes.
Eyup Cagatay Zengin et al. [21] (2019)	25	VLP (25)	18F, 7M	66.6 ± 7.4	RS	16.5 m ± 3.1	Good ROM recovery in flexion–extension and prono-supination with VLP.
Cristoph Bartl et al. [16] (2014)	86	VLP (86)	77F, 9M	74.4 ± 7.1	RCT	12 m	Good clinical outcome with VLP treatment. Malposition of implant was the most frequent complication.
Daniel Martinez-Mendez et al. [10] (2018)	50	VLP (50)	39F, 11M	67 ± 8	RCT	24 m	ROM degree for flexion: 54 ± 13; for extension: 57 ± 11; for supination: 85 ± 8; for pronation: 84 ± 10. The complications were CTS and tendon rupture.
F. Goehre et al. [19] (2014)	40	VLP (21), K-wire (19)	37F, 3M	VLP 71.3 ± 5.7 K-wire 73.8 ± 8.9	RCT	12 m	Slightly better ROM recovery in the VLP group. The VLP group was able to resume daily activities four weeks earlier.
Kristina Lutz et al. [22] (2014)	129	VLP (74), EF (38), K-wire (13)	237F, 21M	74 ± 5	RS	11.3 m ± 9.3	Complication rates: VLP group 22%, EF 42%, and K-wire 23%.
Yu-Yi Huang et al. [9] (2020)	69	VLP (28), EF (41)	59F, 10M	84 (80–97)	RS	EF: 1.3 y ± 0.4 VLP: 1.4 y ± 0.4	Significantly more acceptable radiological parameters at last follow-up in the VLP group The overall incidence of complications was lower in the VLP group.
Brent R Degeorge Jr. et al. [28] (2020)	3740	VLP (3010), EF (257), K-wire (473)	3203F, 537M	74.1 ± 6.4	RS	1 y	The 1-year upper-extremity-specific complication rate was 307.5 per 1000 fractures for operative management.
Mustafa Yalin et al. [17] (2024)	63	VLP (25), EF (25), K-wire (13)		77.25 ± 4.38	RS	1 y	No statistically significant differences in ROM recovery among the three groups.
Kevin C Chung et al. [18] (2020)	187	VLP (65), EF (64), K-wire (58)	163F, 24M	VLP 67 ± 6.2 EF 70 ± 8.4 Wire 68 ± 7.0	RCT	12 m	Better outcomes for extension in the VLP group compared to the EF and K-wire groups.
Rikke Thorninger et al. [26] (2022)	50	VLP (50)	41F, 9M	75 (65.70–80.92)	RCT	12 m	QuickDASH score: 4.2 (−4–+12). PWRE score: 8.6 (2.5–14.7).
Hanna Südow et al. [11] (2022)	33	VLP (33)	32F, 1M	78 (70–90)	RCT	3 y	Flexion–extension arc range (°): 122 ± 19; ulnar deviation (°): 29 ± 5; radial deviation (°): 23 ± 4; radial–ulnar deviation arc (°): 52 ± 6.
Andrew Lawson et al. [24] (2021)	81	VLP (81)	70F, 11M	70.5 ± 7.0	RCT	12 m	The most common was carpal tunnel syndrome on a total of 8% of complications rate.
L P Hung et al. [12] (2015)	26	VLP (26)	21F, 5M	65	HCS	12 m	Flexion (°): 60.0; extension (°): 60.0; supination (°): 90.0; pronation (°): 85.0. No complications were found.
Saeed Ahmed Shaikh et al. [23] (2023)	534	VLP (534)	326F, 208M	64.90 ± 3.70	RCT	1 y	Extension (°): 55.71 ± 10.16; flexion (°): 51.67 ± 4.92; pronation (°): 86.98 ± 4.11; supination (°): 86.68 ± 4.73. Complication: carpal tunnel syndrome.
Muhammad Tahir et al. [13] (2021)	87	VLP (87)	16F, 71M	81 ± 3	RCT	12 m	Extension (°): 62 ± 9; flexion (°): 53 ± 6; pronation (°): 88 ± 2; supination (°): 88 ± 2.
Sondre Stafsnes Hassellund et al. [14] (2021)	50		89F, 27M	73.4 (65–91)	RCT	12 m	Flexion (°): 55; extension (°): 61; ulnar deviation (°): 29; radial deviation (°): 18; supination (°): 83; pronation (°): 82.
Jenny Saving et al. [15] (2019)	58	VLP (58)	55F, 4M	80 (70–90)	RCT	1 y	Extension (°): 55 ± 11; flexion (°): 63 ± 13; supination (°): 96 ± 16; pronation (°): 85 ± 9; ulnar deviation (°): 30 ± 11; radial deviation (°): 22 ± 4.
Giuseppe Solarino et al. [6] (2016)	50	VLP (50)		71 (65–82)	RS	46 m (24–72)	Flexion–extension (°): 81.3 ± 13.4; radial deviation (°): 12.9 ± 3.9; ulnar deviation (°): 25.0 ± 2.0; prono-supination (°): 90.7 ± 7.9.
Marcio Aurelio Aita et al. [27] (2019)	35	EF (35)	24F, 11M	EF 65 (60–73)	RCT	12 m	Good clinical outcome at last follow-up.

## Data Availability

The original contributions presented in the study are included in the article, further inquiries can be directed to the corresponding authors.
